# Scoping review of the emerging definition of long COVID: implications for future research and clinical practice

**DOI:** 10.15446/rsap.V27n6.122127

**Published:** 2025-11-01

**Authors:** Roxana De Las Salas, Maira De la Asunción, Claudia Vásquez-Soto, Kevin Orta-Visbal, Vanessa Serrano, Elizabeth Villarreal, Sharell Sepúlveda, María José Montalvo, Jose Atilio Nuñez, Juana Borja

**Affiliations:** 1 RD: RN. M. Sc. Sciences Pharmacology. Ph. D. Pharmaceutical Sciences. Assistant Professor, Department of Nursing, Universidad del Norte. Barranquilla, Colombia. rdelassalas@uninorte.edu.co Universidad del Norte Department of Nursing Universidad del Norte Barranquilla Colombia rdelassalas@uninorte.edu.co; 2 MA: RN. M. Sc. Nursing. Universidad del Norte. Barranquilla, Colombia. amdelaasuncion@uninorte.edu.co Universidad del Norte Universidad del Norte Barranquilla Colombia amdelaasuncion@uninorte.edu.co; 3 CV: RN. M. Sc. Nursing. Auxiliary Professor, Department of Nursing, Universidad del Norte. Barranquilla, Colombia. claudiav@uninorte.edu.co Universidad del Norte Department of Nursing Universidad del Norte Barranquilla Colombia claudiav@uninorte.edu.co; 4 KO: RN. M. Sc. Nursing. Auxiliary Professor, Department of Nursing, Universidad del Norte. Barranquilla, Colombia. korta@uninorte.edu.co Universidad del Norte Department of Nursing Universidad del Norte Barranquilla Colombia korta@uninorte.edu.co; 5 VS: RN. M. Sc. Nursing. Auxiliary Professor, Department of Nursing, Universidad del Norte. Barranquilla, Colombia. merinod@uninorte.edu.co Universidad del Norte Department of Nursing Universidad del Norte Barranquilla Colombia merinod@uninorte.edu.co; 6 EV: RN. M. Sc. Education. Assistant Professor, Department of Nursing, Universidad del Norte. Barranquilla, Colombia. evillare@uninorte.edu.co Universidad del Norte Department of Nursing Universidad del Norte Barranquilla Colombia evillare@uninorte.edu.co; 7 SS: RN. M. Sc. Nursing. Hospital Universidad del Norte. Barranquilla, Colombia. sharells@uninorte.edu.co Hospital Universidad del Norte Barranquilla Colombia sharells@uninorte.edu.co; 8 MM: RN. M. Sc. Nursing. Hospital Universidad del Norte. Barranquilla, Colombia. jmmontalvo@uninorte.edu.co Hospital Universidad del Norte Barranquilla Colombia jmmontalvo@uninorte.edu.co; 9 JN: MD. Esp. Internal Medicine. M. Sc. Clinical Epidemiology. Assistant Professor, Department of Medicine, Universidad del Norte. Barranquilla, Colombia. anunezj@uninorte.edu.co Universidad del Norte Department of Medicine Universidad del Norte Barranquilla Colombia anunezj@uninorte.edu.co; 10JB: RN. M. Sc. Nursing. Ph. D. Education. Assistant Professor, Department of Nursing, Universidad del Norte. Barranquilla, Colombia. gjuana@uninorte.edu.co Universidad del Norte Universidad del Norte Barranquilla Colombia gjuana@uninorte.edu.co

**Keywords:** Post-acute COVID-19 syndrome, COVID-19, SARS-CoV-2, systematic review *(source: MeSH, NLM)*, Síndrome post agudo de COVID-19, COVID-19, SARS-CoV-2, revisión sistemática *(fuente: DeCS, BIREME)*

## Abstract

**Introduction:**

Long COVID, Post-COVID19 syndrome and prolonged COVID-19, are concepts classified as the set of signs and symptoms that persist after an acute episode of COVID-19 disease.

**Objective:**

To describe what definitions have been published for the term "long COVID".

**Methods:**

The PRISMA ScR (Preferred Reporting Items for Systematic Reviews and Meta-Analyses Extension for Scoping Reviews) was used as a base for a scoping review, as suggested by Joanna Briggs Institute. A search of databases, Medline via PubMed, Embase, SciELO and The Cochrane Library was undertaken. The data registry and synthesis of the results was carried out independently by two reviewers.

**Results:**

Following removal of duplicates, 896 articles were retrieved of which 91 met the eligibility principles and 51 of which included a definition. At least four characteristics of the definitions were identified: time or term, organs affected, symptoms and clinical manifestations.

**Conclusions:**

The review identified many concepts and definitions of "long COVID". These findings show that there is lack of consensus on the definition of long COVID-19.

## RESUMEN

**Introducción:**

El COVID prolongado, el síndrome post-COVID-19 y el COVID-19 prolongado son conceptos clasificados como el conjunto de signos y síntomas que persisten después de un episodio agudo de la enfermedad COVID-19.

**Objetivo:**

Describir las definiciones que se han publicado para el término "COVID prolongado".

**Métodos:**

Se utilizó el PRISMA ScR (elementos de información preferidos para revisiones sistemáticas y metaanálisis, extensión para revisiones de alcance) como base para una revisión exploratoria, tal y como sugiere el instituto Joanna Briggs. Se realizó una búsqueda en las bases de datos Medline a través de PubMed, Embase, SciELO y The Cochrane Library. El registro de datos y la síntesis de los resultados se llevaron a cabo de forma independiente por dos revisores.

**Resultados:**

Tras eliminar los duplicados, se recuperaron 896 artículos, de los cuales 91 cumplían los criterios de elegibilidad y 51 incluían una definición. Se identificaron al menos cuatro características de las definiciones: tiempo o plazo, órganos afectados, síntomas y manifestaciones clínicas.

**Conclusiones:**

La revisión identificó varios conceptos y definiciones de "COVID prolongado". Estos hallazgos indican una falta de consenso sobre su definición.

Coronavirus Disease-19 (COVID-19) was first reported to the World Health Organization (WHO) Country Office in China on December 31 2019 1. and on March 11 2020 was declared the pandemic by The WHO 2.

The high percentage of people who have suffered from COVID-19 have reported, after recovery, a set of clinical manifestations that last beyond 3 weeks and even 3 or more months of symptoms. Three definitions have been developed: acute COVID-19 (0 to 4 weeks), ongoing symptomatic COVID-19 (4 to 12 weeks) and post-COVID-19 syndrome (12 weeks or longer). However, this scoping study focuses on what is related to the long COVID-19 3.

Long COVID or Post-COVID19 syndrome, prolonged COVID-19, are concepts classified as the set of signs and symptoms that persist after an acute episode of COVID-19 disease. Perhaps the most used term is post-COVID19 syndrome 4. Establishing a consensus about a long COVID definition is critical as multiple COVID-19 studies are currently underway internationally, the interventions and outcomes are being defined differently, making it difficult to synthesize the emerging evidence. Clinically the definition of long COVD will have implications for guiding best practice and communication between health care professionals 5.

It should be noted that the persistence of symptoms after coronavirus disease is an entity to determine in the coming years, a marker of the duration of the disease and to establish specific treatment goals 4. The description of the symptoms is relevant in the field of patients who have already been treated and recovered. According to different studies, 20 to 90% of patients who have suffered from COVID-19 present symptoms weeks or months after diagnosis of the infection 4,6.

Therefore, since SARS-CoV-2 infection was recognized in late 2019, the academic and clinical emphasis has been on respiratory manifestations. There is increasing evidence of direct multiorgan effects, and indirect effects on other organ systems and disease processes, such as cardiovascular disease and other chronic conditions, through changes in health care delivery and patient behaviors. Although the long-term effects of COVID-19 on people and health systems are becoming clear, multidisciplinary research is urgently needed 7.

Hence, by elucidating this issue, it will be possible to recognize gaps in the literature and to contribute on the basis of an exploratory definition that demonstrate clinical implications and effects of long COVID-19. The objective of this scoping study was to identify what definitions have been published for the term 'long COVID', and determine whether a unifying definition could be reached.

## METHODS

### Design

The present systematic review was carried out according to the standard protocol of the Joanna Briggs Institute for scoping reviews and was based on the Preferred Reporting Items for Systematic Reviews and Meta-Analyses extension for scoping reviews (PRISMA-ScR) checklist. This study has been registered in the International Platform of Registered Systematic Review and Meta-analysis Protocols INPLASY202290122 (https://doi.org/10.37766/inplasy2022.9.0122).

**Figure 1:**
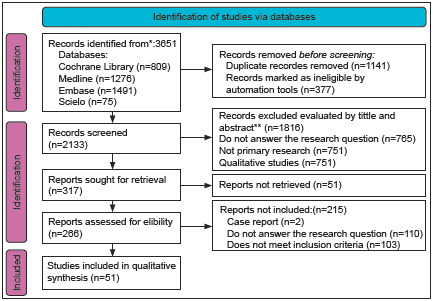
PRISMA flow diagram for new systematic reviews which included searches of databases

The research question was: What are the long COVID-19 concepts and definitions available to incorporate in clinical practice? The PRISMA-ScR flowchart (Figure 1) was adapted.

### Eligibility criteria

Type of studies: randomized clinical trials, quasi-experimental, observational studies, or any type of study that showed evidence regarding concepts, definitions or clinical manifestations of long COVID-19 (Table 1).

**Table 1:** Articles providing an emerging definition of Long COVID

Author (Year)	Country	Funding source	Type of study	Sample	Population	Severity	Setting	Concept / synonyms	Definition	Clinical manifestations/ symptoms (organ affected)
Wurz, 2022 36	Canada	This study was not funded.	Retrospective cohort study	169	Male and female. Ages from 18 to 79	NS	NS	LONG COVID	It is characterized by multiorgan impairments that span respiratory, cardiovascular, neurological, dermatological, and gastrointestinal systems.	Fatigue, shortness of breath, dry cough, cognitive impairment, headache, heart palpitations, chest tightness, and dizziness.
Bourmistrova, 2021 9	United Kingdom	NIHR Maudsley Biomedical Research Centre and Maudsley NHS Foundation Trust in partnership with King's College London	Systematic review	33	NS	NS	NS	COVID Long-term effects	NS	Anxiety, Depression, PTSD, sleep quality disturbance.
Bende, 2021 47	Romania	This research received no external funding.	PILOT STUDY	97	Male and female ages 21-55	Light or moderate symptoms	AMBULATORY	Post-COVID19 Syndrome	Long COVID is referred to manifestations continuing even 12 weeks after.	Persisting symptoms such as fatigue, shortness of breath, chest discomfort, palpitations, and reduced exercise capacity.
Boesl, 2021 51	Germany	German Research Foundation and Open Access Publication Fund of Charité - Universitätsmedizin Berlin.	Retrospective cohort study	100	Male and female 20-79	Mild and severe COVID19	AMBULATORY AND HOSPITALIZED	Post-COVID19 Syndrome	Signs and symptoms that develop during or after an infection with SARS-CoV-2 and continue for more than 12 5weeks and are not explained by an alternative diagnosis.	Neurological and psychiatric symptoms including fatigue, cognitive impairment, insomnia, myalgia, headache, vertigo, anxiety, and depression.
Natarajan, 2022 10	United Kingdom	National Institute for Health Research (NIHR) Research and Southern Health NHS Foundation Trust.	Systematic review	Systematic review	Male and female	N-S	NS	LONG COVID	“Long COVID’ is commonly used to describe symptoms that continue or develop after acute SARS-CoV-2 diagnosis post-4 weeks”	General symptoms: general pain, muscle or joint pain, and mobility dysfunction, fatigue, fever, hair fall, skin rash, and weight loss. Neurological: headache, cognitive impairment, and loss of smell, taste, and hearing. Mental: depression, anxiety, PTSD, and sleep disturbances. Cardiopulmonary symptoms: chest pain, sore throat, dyspnea, palpitations, and cough. Gastrointestinal: poor appetite, diarrhea and emesis, diarrhea or emesis, nausea, and abdominal pain.
Jenning, 2021 5	Switzerland	Science Foundation Ireland COVID-19 Programme-20/COV/8493.	Systematic review	NS	Male and female	NS	INPATIENT / OUTPATIENT	Post-COVID19 Syndrome	‘Post-COVID-19 syndrome’ (PCS), terms that describe persistent signs and/or symptoms in the periods from 4 to 12 weeks and over 12 weeks post-infection onset, respectively.	Fatigue: most common symptom in PCS 44% (10-71%). Dyspnea, myalgia, and sleep disorder prevalent (mean) 40% (6-73%), 34% (2-86%), and 33% (18-57%), respectively. Other symptoms: cough (22%; 3-59%), hair loss (20%; 6-29%), palpitations (20%; 4-62%), arthralgia (13%; 6-29%), throat pain (12%; 3-29%), anosmia (10%; 5-13%), and chest pain (10%; 1-22%). Fever (8%; 1-20%), ageusia 7(8%; 2-15%), and skin problems (6%; 3-12%).
Bliddal, 2021 45	Denmark	Sygesikring Danmark and by the Independent Research Fund Denmark.	Prospective cohort study	129	Male and female	Mild Covid19	NOT HOSPITALIZED	Post-COVID19 Syndrome	“Post-COVID-19-syndrome” for symptoms lasting more than 12 weeks.	Fatigue (16%) and concentration difficulties (13%).
Philip, 2022 11	United Kingdom	Imperial College Clinician Investigator Scholarship	Clinical trial	150	Male and female	N-S	NS	Post- COVID-19	Post-COVID condition refers to symptoms 3 months from initial infection and lasting at least 2 months.	Long COVID is a heterogeneous condition that can involve multiple organs, resulting in numerous, often debilitating symptoms: breathlessness, anxiety, and reduced quality of life.
Huynh, 2022 51	Vietnam	There is no funding to report.	Cross- sectional study	325	Male and female 18-76	N-S	NS	LONG COVID	Long COVID: describe signs and symptoms which continue after acute COVID-19 from 4 to 12 weeks.	Psychological effects including anxiety disorders, depressive manifestations, sleep problems and others.
Malik, 2021 21	United States	N-S	Systematic review	12	Male and female	N-S	NS	Post-Acute COVID-19 Syndrome	Post-acute COVID-19 syndrome (PCS) is defined as an ongoing symptomatic illness in patients who have recovered from their initial COVID-19 infection.	These persistent symptoms include fatigue, dyspnea, anosmia, sleeping difficulties, chest pain, headache, cough, and mental health problems.
Vimercati, 2021 39	Italy	This research received no external funding.	Retrospective cohort study	352	Male and female 20-73	N-S	HOSPITALIZED AND NOT HOSPITALIZED	LONG COVID	Long COVID: persistence of symptoms, or the development of new symptoms, relating to SARS- CoV-2 infection late in the course of COVID-19, at least 28 days after diagnosis.	Typical symptoms: dyspnea, tachycardia and extreme fatigue, psychopathological symptoms related to intense distress (i.e., PTSD, secondary traumatic stress, complicated grief and anxiety, myalgia, muscle fatigue amongst others).
Nowakowski, 2022 22	United States	National Institutes of Health (NIH); the Department of Veteran Affairs, Veterans Health Administration, Office of Research and Development; and the Center for Innovations in Quality, Effectiveness and Safety.	Retrospective cohort study	74	Male and female	Mild and severe COVID19	HOSPITALIZED AND NOT HOSPITALIZED	Post- COVID-19	Post COVID-19 conditions occur in individuals with a history of probable or confirmed SARS CoV-2 infection, usually 3 months from the onset of COVID-19 with symptoms, and last for at least 2 months and cannot be explained by an alternative diagnosis.	Sleep disturbances in more than 4 out of 10 individuals. Depression and anxiety in more than 3 in 10 individuals following the resolution of acute symptoms. Poor sleep quality following an acute COVID-19 infection is associated with female gender, medical comorbidities, and indicators of disease severity including number of symptoms on admission, duration of hospital stay, necessity of mechanical ventilation, and hypertension requiring vasopressors.
Tauekelova, 2022 55	Kazakhstan	Science Committee of the Ministry of Education and Science of the Republic of Kazakhstan.	Prospective cohort study	312	Male and female Median age 54	Mild and severe COVID19	HOSPITALIZED AND NOT HOSPITALIZED	Long covid and Post-COVID19	Long-COVID-19 or post-COVID-19 syndrome describes symptoms lasting for more than three months after the first COVID-19 symptoms onset.	Fatigue (220, 70.51%), tiredness (180, 57.69%), and sleep disturbances (168, 53.85%), muscle pain (109, 34.9%), memory disfunction (108, 34.9%), dizziness (34.9%), headache (79, 25.3%), instability of blood pressure mostly with increased systolic blood pressure (62, 19.9%), palpitation (47, 15.1%), dyspnea on exertion (45, 14.4%), joint pain (39, 12.5%), and increased sweating (39, 12.5%).
Díaz-Salaza, 2022 28	Spain	N-S	Case-control study	121	Male and female 18-88	Mild Covid19	Primary care	Post-COVID19 Syndrome	Signs and symptoms that develop during or after an infection consistent with COVID-19, that last more than 12 weeks, and that are unexplained by an alternative diagnosis.	These symptoms include fatigue -the most frequent-, myalgia, dyspnea, anosmia/ageusia, autonomic dysregulation manifested as orthostatic hypotension, tachycardia, thermoregulation or gastrointestinal disturbances, and cognition alterations, leading to a significant impact on the quality of life.
Giurgi-Oncu, 2021 48	Romania	This research received no external funding.	Prospective cohort study	143	Male and female 18-55	Mild and severe COVID19	NS	Post-Acute COVID-19 Syndrome	Post-acute COVID-19 syndrome, characterized by persisting symptoms up to 12 weeks after the acute illness.	Residual symptoms, such as fatigue, dyspnea/persistent oxygen requirement, chest pain, post-viral chronic malaise, headaches, neurocognitive (brain fog), and mental health difficulties, such as anxiety, depression, disturbed/ nonrestorative sleep, or psychotic episodes.
Décary, 2021 37	Canada	Alberta Health Services, the SPOR Evidence Alliance (SPOR EA), and the COVID-19 Evidence Network to support Decision-making (COVID-END).	Systematic review	N-S	Male and female	N-S	HOSPITALIZED AND NOT HOSPITALIZED	LONG COVID	Long COVID translates into symptoms that develop during or following an infection from COVID-19 and continue for 4 weeks or more.	N-S
Michel, 2021 12	United Kingdom	This work was supported by the UK Foreign, Commonwealth and Development Office and Welcome, the Bill & Melinda Gates Foundation and the EU FP7 project PREPARE.	Systematic review	10.951	Male and female	Mild and severe COVID19	HOSPITALIZED AND NOT HOSPITALIZED	Post-COVID19 Syndrome	Post COVID-19 syndrome continuing for over 12 weeks	The most common were weakness, general malaise, fatigue, concentration impairment and breathlessness. Patients also reported a diverse array of less prevalent symptoms and signs, including sweating, chest pain, sore throat, anxiety and headaches, among others.
Davis, 2021 13	United Kingdom	AA's research grant (Welcome Trust/ Gatsby Charity via Sainsbury Welcome center, UCL).	Prospective cohort study	3762	Male and female 18-80+	Mild and severe COVID19	HOSPITALIZED AND NOT HOSPITALIZED	LONG COVID	A collection of symptoms that develop during or following a confirmed or suspected case of COVID-19, and which continue for more than 28 days.	Fatigue, post- exertional malaise, and cognitive dysfunction. Symptoms varied in their prevalence over time, and we identified three symptom clusters, each with a characteristic temporal profile.
Meza-Torres, 2022 14	United Kingdom	The Imperial College President’s Excellence Fund, the Economic and Social Research Council, UK Research and Innovation, Health Data Research UK, the NIHR Imperial Biomedical Research Centre, the NIHR Oxford Biomedical Research Centre, and the NIHR Imperial Patient Safety Translational Research Centre.	Retrospective cohort study	416505	Male and female, 44.5 (SD 21.7) years	Community and Hospital	Ambulatory	LONG COVID	Long COVID is defined as fatigue, breathlessness, cognitive dysfunction, and a variety of other symptoms occurring after COVID-19 infection.	Central nervous system (Memory loss and confusion, Trouble sleeping, Difficulty concentrating, Loss of smell and taste, Vertigo and dizziness), Respiratory (Sore throat, Shortness of breath, Cough), Cardiovascular (Palpitations, chest pain), Gastrointestinal (Nausea and vomiting, Loss of appetite, Diarrhea), Mental health (Worry and anxiety, Low mood and not enjoying anything), General (Weakness and tiredness, Fever, Muscle aches, Abdominal pain).
Hurk, 2022 23	United States	The University of Dayton STEM Catalyst Initiative and NIMH and the Pulmonary Wellness Foundation.	Retrospective cohort study	338	Male and female	Mild and severe COVID19	H and NH	LONG COVID	Symptoms persist for three months or more.	The syndrome is highly variable in presentation, although fatigue, dyspnea, cough, chest pain, headache, chemosensory impairment, diarrhea, and muscle pain are reported most frequently.
Twomey, 2022 38	Canada	The O’Brien Institute of Public Health and Ohlson Research Initiative, Cumming School of Medicine, University of Calgary and Canadian Institutes of Health Research Fellowship.	Cross- sectional study	213	Male and female 18-79	Mild and severe COVID19	NS	LONG COVID	Post COVID-19 condition occurs in individuals with a history of probable or confirmed SARS-CoV-2 infection, usually 3 months from the onset of COVID-19 with symptoms that last for at least 2 months and cannot be explained by an alternative diagnosis (WHO definition).	Common symptoms include fatigue, shortness of breath, and cognitive dysfunction, but there are also others that generally have an impact on everyday functioning.
Jimeno- Almazán, 2022 29	Spain	Hospital Médico Virgen de la Caridad and the cardiology clinic Cardiosalus	Cross- sectional study	32	Male and female mean age of 45 years	Mild Covid19	AMBULATORY	Long covid and Post-COVID19	Persistence of clinical manifestations lasting more than 12 weeks and cannot be explained by an alternative diagnosis.	The post-COVID-19 condition patients mostly refers to fatigue, post- exertional malaise, dyspnea, headache, and many other neurocognitive conditions described as brain fog or inability to perform daily physical tasks.
Evans, 2021 15	United Kingdom	UK Research and Innovation and National Institute of Health Research	Prospective cohort study	2320	Male and female, mean age 58·7	‘very severe’, ‘severe’, ‘moderate/ cognitive’, and ‘mild’	HOSPITALIZED	LONG COVID	NICE definition / World Health Organization	Fatigue, aching muscles, physical slowing down, poor sleep, breathlessness, joint pain or swelling, slowing down in thinking, pain, short term memory loss and limb weakness (after one year).
Pfaff, 2022 24	United States	National Institutes of Health (NIH).	Retrospective cohort study	21072	Male and female	NS	NS	LONG COVID	LC is defined by ongoing, relapsing, or new symptoms or other health effects occurring after the acute phase of SARS- CoV-2 infection (i.e., present four or more weeks after the acute infection).	Heterogeneous symptoms may include, but are not limited to, fatigue, difficulty breathing, brain fog, insomnia, joint pain, and cardiac issues.
Jennings, 2022 54	Ireland	Science Foundation Ireland (SFI)	Cross- sectional study	108	Male and female mean age of 46.3 years	Mild Covid19	HOSPITALIZED AND NOT HOSPITALIZED	LONG COVID	LC can be defined as signs and symptoms that persist or develop past the acute phase that cannot be explained by an alternative diagnosis.	Characterized by multisystem dysfunction, with fatigue, dyspnea, sleep disorder, and myalgia among the most prevalent long- term symptoms.
Guo, 2022 16	United Kingdom	Department of Psychology, University of Cambridge.	Cross- sectional study	421	Male and female aged 18 and over	Mild and moderate COVID19	NS	Long covid and Post-COVID19	“Signs or symptoms that develop during or after infection consistent with COVID19, continue for more than 12 weeks and are not explained by an alternative diagnosis”.	Cognitive symptoms (77.8% difficulty concentrating, 69% brain fog, 67.5% forgetfulness, 59.5% ToT word-finding problems, and 43.7% semantic disfluency (saying or typing the wrong word). Chronic fatigue like (“Fatigue/Mixed”). “CP/ Fatigue,” “Neurological” and “Gastrointestinal/ Autoimmune” symptoms.
Martínez-Salazar, 2022 33	Switzerland	The Swiss National Science Foundation (SNSF)	Review study	N-S	N-S	ANY SEVERITY	NS	LONG COVID SYNDROME	LCS includes a number of different terms such as “Post-acute COVID-19” and “Post-COVID-19 syndrome”. “Post- acute COVID-19″, describing patients who still have symptoms after 4-12 weeks, while patients with symptoms after more than 12 weeks are classified under the “Post-COVID-19 syndrome”.	Fatigue, headache, attention deficit, hair loss, and shortness of breath, chest pain, palpitations, tachycardia, depression and neurologic impairment and dysfunction. Cardiovascular: chest pain is the most described symptom in patients with prior COVID-19 regardless of severity. Palpitations are also reported with a frequency of about 10% two to six months after COVID-19 diagnosis, and heartbeat irregularities. Vasculature: pulmonary and extrapulmonary thromboembolism.
Johnsen, 2021 46	Denmark	N-S	Cross- sectional study	57	Male and female	N-S	H and NH	LONG COVID	NS	Breathlessness, cough and fatigue. Neurological: loss of smell and taste, tingling sensations, dizziness and severe fatigue, cognitive impairments for months after their recovery.
Jamoulle, 2022 49	Belgium	The study is financed from own funds.	Descriptive and Narrative Study	34	Male and female mean age 40	ANY SEVERITY	HOSPITALIZED AND NOT HOSPITALIZED	LONG COVID	Long Covid refers to symptoms persisting for more than four weeks after the diagnostic, usually managed in general practice.	Unbearable fatigue, brain fog and myalgia. Cognitive disorders, memory and attention deficits, anomia, dysarthria, frontal behavioral disorders, autonomic dysregulation, headaches, dyspnea, anosmia, dysgeusia, skin or digestive disorders, psychosocial distress, loneliness, anxiety, depression and sleep disorders.
Yamamoto, 2022 41	Japan	This research did not receive any specific grant from funding agencies in the public, commercial, or not- for-profit sectors.	Retrospective cohort study	39	Male mean age LOH group was 36,0 and non-LOH group was 38,5	ANY SEVERITY	HOSPITALIZED AND NOT HOSPITALIZED	Long covid and Post-COVID19	These persistent sequelae, which have been termed “post COVID-19 condition” by the World Health Organization.	Fatigue, dysgeusia, dyssomnia, low-grade fever, headache, and alopecia. Male hypogonadism, called late-onset hypogonadism (LOH): impaired production and secretion of testosterone can directly deteriorate accompanying fatigue and metabolic syndrome in addition to sexual impotency.
Hughes, 2022 17	United Kingdom	National Institute for Health Research (NIHR) and UK Research and Innovation (UKRI)	Mixed study	274	Male and female mean age 45,1	Mild and moderate COVID19	NOT HOSPITALIZED	Long covid and Post-COVID19	These persistent symptoms which have been termed “post COVID-19 condition” by the World Health Organization.	Fatigue, dyspnea, and impaired concentration. Symptoms may be persistent, cyclical, or episodic with negative consequences for work capability, functioning, and quality of life.
Rezel-Potts, 2021 18	United Kingdom	NIHR Biomedical Research Centre at Guy’s and St Thomas’ NHS Foundation, King’s College London and e British Heart	Retrospective cohort study	372816	Male and female mean age 33	ANY SEVERITY	HOSPITALIZED AND NOT HOSPITALIZED	LONG COVID	LC: symptoms persisting more than 12 weeks. It is often self-identified by patients with the concept focusing on symptom burden and impacts on quality of life.	Cardiovascular: pulmonary embolism, atrial arrhythmias and venous thromboses.
Rass, 2022 43	AUSTRIA	Land Tirol GZ / Boehringer Ingelheim	Retrospective cohort study	906	Male and female mean age 45	Mild, moderate and severe COVID-19	HOSPITALIZED AND NOT HOSPITALIZED	Post- COVID19, Long covid and post-acute sequelae of COVID-19 (PASC)	All post-COVID-19 symptoms over 28 days are subsumed under ‘long COVID’. Persistent symptoms present for > 12 weeks were coarsely classified as PASC.	Post-acute symptoms: fatigue, respiratory and neurocognitive complaints. Despite a protracted smell and taste dysfunction, this subset had high ratings of physical performance, mental health, and quality of life.
McNarry, 2022 20	United Kingdom	Welsh Government / Higher Education Funding Council for Wales Research Wales Innovation Fund / Centre for Physical Activity Research (TrygFonden)	Clinical trial	147	Male and female over 18 years old	N-S	NS	LONG COVID	The persistence of COVID-19-related symptoms was noted in May 2020. LC: defined as ongoing or new symptoms ≥4 weeks post- infection.	One of the top three most debilitating symptoms associated with a poor quality of life was dyspnea (breathlessness).
Antoniou, 2022 52	Greece	European Respiratory Society	Systematic review	143	Male and female	N-S	NS	Post-COVID19 Syndrome (PCS)	PCS: "signs and symptoms that develop during or after an infection consistent with COVID-19, which continue for more than 12 weeks and are not explained by an alternative diagnosis".	The main persistent symptoms reported in the included studies were fatigue (50-65% of patients) and anxiety/ depression (20-40%).
Delgado- Alonso, 2022 30	Spain	Department of Health of the Community of Madrid. Instituto de Salud Carlos III / European Regional Development Fund	Clinical trial	93	Male and female mean age 50,39	N-S	NS	Post-COVID19 Syndrome	WHO: PC condition or PCS is a disorder occurring in patients with a history of n-SARS-CoV-2 who present symptoms that cannot be explained by an alternative diagnosis.	Common symptoms: fatigue, shortness of breath, and cognitive dysfunction. Other common symptoms: depression, anxiety, headache, joint and muscle pain, sleep problems, and smell or taste disorders.
König, 2022 44	AUSTRIA	University of Vienna	Clinical trial	60	Male and female	NS	NOT HOSPITALIZED	LONG COVID and Post- COVID19 Syndrome	PCS or LC: presence of symptoms longer than 3 months after the infection with SARS-CoV-2.	Fatigue, reduced physical capacity, dyspnea, ageusia, anosmia, musculoskeletal pain and neuropsychological (depression, anxiety, insomnia and a loss of concentration).
Cattadori, 2022 40	ITALY	Ministry of Health- Ricerca Corrente / IRCCS MultiMedica.	Review study	N-S	N-S	N-S	NS	LONG COVID SYNDROME	LCS: Most patient are discharged without breathlessness at rest, yet often with poor exercise tolerance associated with persistency of COVID-19 signs at RX or CT pre-discharge evaluation.	Respiratory function: pulmonary fibrosis in the long run. Cardiac: possible persistent myocardial damage in the long run. Pulmonary vessels: pulmonary hypertension in some cases due to pulmonary embolism and/or thrombosis. Decreased exercise capacity is the most common dysfunction (61,4% patients) mainly due to the long-term immobilization.
Aparisi, 2021 31	Spain	Castilla and León Regional Health Authority / Spanish Society of Cardiology	Prospective cohort study	70	Male and female mean age 54,8	Mild, moderate and severe COVID	H and NH	Post-COVID19 Syndrome	NS	Lung conditions, such as acute pulmonary embolism, pneumonia, chronic interstitial lung diseases, and after viral infections.
Diem, 2022 34	Switzerland	The author(s) received no financial support for the research, authorship, and/or publication of this article.	Retrospective cohort study	42	Male and female mean age 44,8	Mild, moderate and severe COVID-19	HOSPITALIZED AND NOT HOSPITALIZED	LONG COVID and Post- COVID19 Syndrome	PC, LCS, PCS: signs and symptoms that develop during or after an infection consistent with COVID-19, continue for >12 weeks and are not explained by an alternative diagnosis”.	The three most prevalent PC symptoms were fatigue (38/42, 90.5%), depression (22/42, 52.4%) and sleep disturbance (20/42, 47.6%). Sleep disturbance consisted of insomnia including difficulties in initiating (13/20, 65%) and maintaining sleep (7/20, 35%).
Fernández-De-las-peñas, 2022 32	Spain	Novo Nordic Foundation (Denmark) / European Regional Development, Cohesion Fund, REACT-EU	Prospective cohort study	1969	Male and female mean age 61,1	Mild and severe COVID19	HOSPITALIZED	Post- COVID-19	NS	Fatigue (61.3%) and dyspnea at exertion (53.5%). The number of post COVID symptoms was significantly higher (p < 0.001) in females (mean: 2.25, SD: 1.4) than in males (mean: 1.5, SD: 1.3).
Long, 2021 50	China	The analysis was supported by the Novel Coronavirus Prevention and Treatment Emergency Scientific Research Project of Xiamen University	Systematic review	4.478	Male and female mean age 50-60	Mild, moderate and severe COVID-19	HOSPITALIZED AND NOT HOSPITALIZED	Long COVID-19 and post-acute COVID-19	LC or PACS is defined as persistent signs and symptoms that emerge during or after SARS-CoV-2 infection, usually lasting>4 weeks and with all other possible diagnoses excluded.	CPS: chest pain, dyspnea, cough, sore throat, palpitation, and chest distress. N: memory impairment, cognitive impairment, headache, taste disorder, and smell disorder. MS: myalgia and joint pain. GI: diarrhea or vomiting, abdominal pain, and decreased appetite. Psychosocial: PTSD, anxiety or depression, attention deficit disorder, sleep difficulties, and hair loss. Fatigue or weakness, skin rash, fever, pain, discomfort, and dizziness.
Carter, 2022 25	United States	Indiana Clinical and Translational Sciences Institute	Case-control study	32	Female mean age 54,5	NS	NS	Post-Acute COVID-19 Syndrome	PACS: Some individuals experience a delayed recovery with symptoms persisting three to 4 weeks (or longer) beyond initial SARS- CoV-2 diagnosis-a condition now recognized.	Among these individuals, latent effects can vary considerably but generally include dyspnea, fatigue, mental fog and/ or sensory disturbances.
Reese, 2023 26	United States	National Institutes of Health /National Institutes of Health Center of Excellence in Genome Sciences / Donald A. Roux Family Fund at the Jackson Laboratory. Marsico Family at the University of Colorado Anschutz	Retrospective cohort study	2466	Male and female mean age 51,9	NS	HOSPITALIZED AND NOT HOSPITALIZED	Post- COVID-19 condition	WHO PCC: diagnosed several months after the onset of acute symptoms of COVID-19 based on new-onset or lingering symptoms which cannot be explained by an alternative etiology and continue for at least two months.	Multi-system symptoms including fatigue, post-exertional malaise, dyspnea, cough, chest pain, palpitations, headache, arthralgia, weakness (asthenia), paresthesias, diarrhea, alopecia, rash, impaired balance, and memory or cognitive dysfunction.
Chen, 2022 27	United States	University of Michigan School of Public Health, Center for Precision Health Data Science, the Michigan Institute for Data Science, the National Science Foundation / National Institutes of Health.	Meta-Analysis and Systematic Review	33	Male and female	Mild, moderate and severe COVID-19	HOSPITALIZED AND NOT HOSPITALIZED	LONG COVID	The condition that occurs in individuals with a history of probable or confirmed SARS-CoV-2 infection, usually 3 months from the onset of COVID-19, with symptoms that last for at least 2 months and cannot be explained by an alternative diagnosis.	The 5 most prevalent symptoms were the following: Fatigue, memory problems, dyspnea, sleep problems, and joint pain. Neurologic: Memory problems, Sleep problems, Concentration/ confusion/ brain fog, Taste, Smell, Headache. Respiratory Symptoms: Dyspnea, Cough, Chest pain. Psychological Symptoms: Anxiety, Depression. MS: Joint pain, Myalgia. GI: Abdominal pain, Diarrhea. Dermatologic Symptoms: Hair loss.
Paul, 2022 19	United Kingdom	The Nuffield Foundation/ Mental Health Network funded by the Cross-Disciplinary Mental Health Network Plus initiative supported by UK Research and Innovation and the Welcome Trust	Cross- sectional study	1581	Male and female 18-59	Mild, moderate and severe COVID-19	HOSPITALIZED AND NOT HOSPITALIZED	LONG COVID and Post- COVID19 Syndrome	LC: which includes both ongoing symptomatic COVID-19 (the presence of symptoms from 4 to 12 weeks post-onset), and PCS (the presence of symptoms > 12 weeks post-onset).	The most common symptoms are weakness, fatigue, cognitive difficulties (e.g., concentration and remembering), and breathlessness.
Shah, 2022 53	India	This research did not receive any specific grant	Prospective cohort study	212	Male and female mean age 50,6	Mild, moderate and severe COVID-19	HOSPITALIZED AND NOT HOSPITALIZED	PACS or “Long Haul COVID-19”	They characteristically occur within 3 and 12 weeks post recovery and are labelled as Post- acute COVID-19 syndrome or “Long Haul COVID-19”	Although not well characterized, common presenting symptoms pertaining to the cardiovascular system include fatigue, dyspnea, chest pain, orthostatic intolerance, lightheadedness and palpitations.
Sunada, 2022 42	Japan	NS	Retrospective cohort study	186	Male and female mean age 40	Mild, moderate and severe COVID-19	HOSPITALIZED AND NOT HOSPITALIZED	LONG COVID	Long COVID was defined as symptoms that persist for more than one month after the onset of COVID-19.	General symptoms: general malaise, dysgeusia, dyssomnia, low-grade fever, headache, hair loss, alopecia, dyspnea, and sleepless- ness. Fatigue and dyssomnia/dysgeusia were more frequent in younger patients, while hair loss was more frequent in older female patients.
Kjellberg, 2022 57	Sweden	The Swedish Heart- Lung foundation, Stockholm Health Council and Oura Health Oy.	Clinical trial	80	Male and female 18-60	NS	NS	LONG COVID	LC: symptoms persist 12 weeks after the initial SARS-CoV-2-infection, is a substantial problem for individuals and society in the surge of the pandemic.	Long- term symptoms: shortness of breath, fatigue, post-exertional malaise, and cognitive dysfunction (reduce working capability). Some patients are also diagnosed with autonomic dysfunction, including POTS and inappropriate sinus tachycardia.
Becker, 2021 35	Switzerland	The Swiss National Science Foundation / The Gottfried and Julia Bangerter- Rhyner Foundation	Prospective cohort study	90	Male and female mean age 60,09	MODERATE AND SEVERE COVID-19	HOSPITALIZED	LONG COVID	Long COVID has been defined as residual symptoms after acute disease, which persist for more than 4 weeks.	Fatigue 18 (28%), Dyspnea 9 (14.29%), Concentration difficulties 9 (14.29%), Joint pain 6 (9.5%), Post-exertion malaise.
Chudzik, 2022 56	Poland	Wroclaw Medical University	Retrospective cohort study	2218	Male and female. Age(±SD)= 53.8 ± 13.5	Mild, moderate, severe COVID-19	Out patient	LONG COVID	The severity of COVID-19 increased the risk of developing long- COVID syndrome.	Chronic fatigue, Headache, Cought, Brain fog, Dyspnoea, Hair loss, Olfactory dysfunction, Osteoarticular pain.

NS: Non specify. PTSD: Post-traumatic stress disorder. LC: Long COVID. PC: Post COVID-19. PCS: Post-COVID-19 syndrome. PCC: post COVID-19 condition. LCS: Long-Covid Syndrome. PACS: post- acute COVID-19 syndrome. H: HOSPITALIZED. NH: NOT HOSPITALIZED. ToT: tip-of-the tongue. PASC: post-acute sequelae of COVID-19. LOH: late-onset hypogonadism. n-SARS-CoV-2: novel severe acute respiratory syndrome coronavirus 2. WHO: World Health Organization. RX: X ray. CT: computerized tomography scan. CPS: cardiopulmonary system. N: Neurological. MS: Musculoskeletal. GI: Gastrointestinal. POTS: Postural Orthostatic Tachycardia Syndrome

### Search methods for study selection

Databases searched was conducted between 2015 and 2022 and included: Medline via PubMed, Embase, Scielo and The Cochrane Library. Additionally, a manual search was conducted. The search algorithm used was composed of free terms and indexed according to each database (Quadro 1).

**Quadro 1:** Search strategy and syntax by database

Database	Keyword
Medline via Pubmed	(("Adult"[Mesh]) AND ( "COVID-19"[Mesh] OR "SARS-CoV2"[Mesh] OR "post-acute COVID-19 syndrome" [Supplementary Concept] )) OR ( "SARS-CoV-2 variants" [Supplementary Concept] OR "COVID-19 post-intensive care syndrome" [Supplementary Concept] )) AND ( "Models, Nursing"[Mesh] OR "Outcome and Process Assessment, Health Care"[Mesh] OR "Needs Assessment"[Mesh] OR "Patient Outcome Assessment"[Mesh] OR "Symptom Assessment"[Mesh] OR "Health Impact Assessment"[Mesh] OR "Nursing Assessment"[Mesh] )
EMBASE	#1 'long covid'/exp OR 'covid long-hauler' OR 'covid-19 longhauler' OR 'chronic covid syndrome' OR 'chronic covid-19' OR 'long covid' OR 'long haul covid' OR 'long hauler covid' OR 'post covid 19 fatigue' OR 'post covid 19 neurological syndrome' OR 'post covid 19 syndrome' OR 'post covid fatigue' OR 'post covid impairment' OR 'post covid syndrome' OR 'post-acute covid syndrome' OR 'post-acute covid-19' OR 'post-acute covid-19 fatigue' OR 'post-acute covid-19 neurological syndrome #2 AND ([adult]/lim OR [aged]/lim OR [very elderly]/lim) (#1 AND #2)
SciELO	adult AND ((covid) AND (sars-cov-2)) AND (long covid) OR (post acute covid) AND NOT (children) AND NOT (animals).
THE COCHRANE LIBRARY - CENTRAL	post-acute and COVID-19 and syndrome

### Study selection and data extraction

One researcher (MA) screened the results by title and abstract looking for potential studies. Two authors (MA and RD) independently identified all occurrences of the term Long COVID within the included articles and extracted the data using a standardized data collection sheet (Table 1). The data collection sheet was developed and piloted by the first author (RD) and then piloted by the second author (MD) who found it usable, and no further changes were made.

Descriptive analysis was conducted extracting the following data: Country, founding source, type of study, sample, study population, COVID-19 severity, environment of care, Concept/Synonyms, definition (long COVID-19), clinical manifestations/symptoms (organs affected). A search strategy was performed in the selected databases.

A comparison of the quality was not feasible due to the inclusion of numerous study types (e.g., original research and non-systematic reviews).

### Synthesis of results

Conventional content analysis was used to review Concept/Synonyms, definition, clinical manifestations/ symptoms (organs affected). The data collected were synthesized in a table that includes the information found in the studies for each category and the information is presented such as meta-summary.

For reporting the results and preparing the manuscript, the PRISMA-ScR scope review checklist was followed 8.

### Ethical considerations

Since it was a scoping review that did not involve humans, it was not necessary to get an Ethics Committee's approval.

## RESULTS

From the search, a total of 3 651 articles were found. A total of 970 were duplicates and were discarded. After screening title and abstract, 1 216 articles were eliminated as they did not follow inclusion criteria. A total of 267 articles were retrieved and reviewed in full text. Finally, a total of 51 articles were included in the narrative synthesis. The study selection process is shown in a PRISMA diagram (Figure 1).

### Characteristics of included studies

Regarding the study designs, five were clinical trials 11,20,30,44,57, 30 were cross-sectional studies, 14 were retrospective and 17 prospective. Ten were systematic review among others. The characteristics of the studies are presented (Table 1).

Most of the articles were published between 2021 and 2022. A considerable number of concepts/synonym and definitions for "long COVID-19" reported in the literature. The most frequent environment of care was ambulatory and hospitalized (outpatient / Inpatient) (Table 1).

### Context of development of the studies

From a total of 51 studies, 3 were conducted inpatient and 8 outpatients. Twenty three enrolled inpatient and outpatient. Seventeen were not specified.

### Concept/synonym and definition

The synonymous terms mostly reported in the literature were long COVID, Post-COVID19, Post-acute-COVID-19 syndrome, Post-COVID19 Syndrome, among others (Table 1).

### Duration time of long COVID-19

The definition most used and referenced by a large number of studies is "Signs and symptoms that develop during or following an infection consistent with COVID-19, continue for more than 12 weeks and are not explained by an alternative diagnosis" (Table 3) 3.

### Clinical manifestations/symptoms (organs affected)

This includes neurologic and psychiatric, musculoskeletal, and respiratory system symptoms.

Commonly related symptoms include fatigue, shortness of breath, dry cough, cognitive impairment, insomnia, myalgia, vertigo, anxiety, headache, heart palpitations, chest tightness, dizziness and depression 3. Other studies included this clinical manifestations and also expanded their own symptoms and organs affected 59.

## DISCUSION

This review represents a comprehensive scoping study of evidence regarding long COVID for health care professionals. To effectively diagnose, treat and manage a long COVID-19 condition is needed to define and distinguish the clinical manifestations, system or organs affected, and duration time of long COVID-19.

This review of 51 studies from diverse geographical contexts provides a comprehensive overview of the clinical characteristics, research methodologies, and conceptual definitions about Long COVID-19. While there is growing evidence of the significance of persistent symptoms following acute SARS-CoV-2 infection, substantial heterogeneity remains in study design, populations, and definitions applied. Establishing a consensus about a long COVID definition is critical as multiple COVID-19 studies are currently underway internationally and the interventions and outcomes are being defined differently, making it difficult to synthesize the emerging evidence.

Observational designs were the most frequent type of study 36,47,51. This methodological limitation may constrain the generalizability of findings and underscores the need for more rigorous, prospective research on this topic. Additionally, a significant number of studies reported their funding sources 15,29,38.

Fatigue, cognitive impairment ("brain fog"), dyspnea, and psychological manifestations are the most frequently reported Long COVID symptoms 36,47,51. These findings align with prior work by Dennis et al. 15 and the NICE guidelines 3. However, the variability in terminology-ranging from "Long COVID" to "Post-COVID-19 Syndrome" and "Long-term effects"-reflects ongoing conceptual consensus in the field 9,10,47. This scoping review contribute to a better understanding of the clinical manifestations.

Moreover, only a subset of studies included information on the sex and age distribution of their populations. There was a balanced gender representation, and most participants were adults between 20 and 79 years of age 36,47,51. Pediatric population were not represented. It indicates a gap in current research that should be addressed, given the differential immune and psychosocial responses across age groups.

The settings of care, ranging from ambulatory to hospitalized settings. Studies with inpatient cohorts tended to report more severe or multisystemic manifestations 10,51. This suggests that the clinical manifestations of Long COVID may be modulated by the severity of the acute phase, although further data are needed.

In terms of conceptual clarity, a notable proportion of articles used the NICE definition of Long COVID. Others did not use any operational concept, but mentioned clinical manifestations. This further highlights the urgent need for standardizing diagnostic criteria to facilitate clinical care, public health planning, and comparative research. Bende et al. 47 uses the term 'Post-COVID19 Syndrome' and 'Long COVID' to refer to manifestations continuing even 12 weeks after having been proposed. While Jennings et al. 54 refers to 'post-COVID-19 syndrome' (PCS), term that describe persistent signs and/or symptoms in the periods from 4 to 12 weeks and over 12 weeks post-infection onset, respectively. Similar to NICE 3 definition.

There is currently no long-term evidence base to determine how long the ongoing effects currently seen after a COVID-19 infection will last. The term 'post' COVID-19 syndrome was agreed to reflect that the acute phase of the illness has ended, not that the person has recovered. Because it is not clear how long symptoms may last, NICE 3 suggested to avoid time specific terms such as 'chronic' or 'persistent'. On the other hand, the word 'Syndrome' was accepted to reflect the 'running together' or concurrence of the multisystem/multiorgan, fluctuating and often overlapping 'clusters' of symptoms 3.

Although the evidence base for Long COVID is rapidly expanding, future research must prioritize methodological standardization, longitudinal follow-up, and inclusion of diverse demographic groups to improve our understanding of Long COVID.

On the other hand, specific clinical diagnostic criteria are needed to support health care professionals and the basis of planning services. Being able to identify related conditions will allow to prioritize interventions 60 .

A variety of concepts/synonym and definitions has been distinguished to define 'long COVID'. This scoping study summarizes published evidence of long COVID-19 Concepts/synonym and definitions, associated symptoms and organ system affected. Reaching a consensus on a standard definition for 'long COVID' is essential to research on clinical practice.

This article proposes a definition based on all those used in academic literature to the time of this review: Long COVID can be defined as a set of clinical manifestations that last beyond 12 weeks after the diagnosis of a confirmed COVID-19 and are not attributed to other pre-existing diseases. There is an urgent need for well-designed tools to support health care professionals in managing patients with Long COVID ♠
